# Versatile and Accessible Magnetic Diagnosis Platform with Different Types of Magnetic Particles for Liquid and Solid Biopsies

**DOI:** 10.3390/ijms241210363

**Published:** 2023-06-20

**Authors:** Ju-Fang Liu, Jean-Hong Chen, Shu-Hsien Liao, Kuen-Lin Chen, Wen-Chun Wei, Ting-Yuan Chen, Jen-Jie Chieh, Kai-Wen Huang

**Affiliations:** 1 School of Oral Hygiene, College of Oral Medicine, Taipei Medical University, Taipei 110, Taiwan; 2 Department of Materials Engineering, Kun Shan University, Tainan 710, Taiwan; 3 Institute of Electro-Optical Engineering, National Taiwan Normal University, Taipei 116, Taiwan; shliao@ntnu.edu.tw (S.-H.L.);; 4 Department of Physics, National Chung Hsing University, Taichung 402, Taiwan; 5 Graduate Institute of Clinical Medicine, College of Medicine, National Taiwan University, Taipei 100, Taiwan; 6 Department of Surgery, National Taiwan University Hospital, Taipei 100, Taiwan; 7 Hepatitis Research Center, National Taiwan University Hospital, Taipei 100, Taiwan

**Keywords:** liquid and solid biopsy, magnetic particles, acoustic type of vibration sample magnetometer, soft and hard magnetic types

## Abstract

The diagnosis of liquid and solid biopsies by different instruments makes the clinic loading difficult in many aspects. Given the compositions of magnetic particles (MPs) with diverse characterizations and the innovative acoustic type of vibration sample magnetometer (VSM), the versatile, accessible magnetic diagnosis platform was proposed to meet clinical demands, such as low loading for multiple biopsies. In liquid biopsies of alpha-fetoprotein (AFP) standard solutions and subject serums, molecular concentration was analyzed from saturation magnetization by the soft type of Fe_3_O_4_ MPs with AFP bioprobe coating. In the phantom mixture simulated as bounded MPs in tissue, the bounded MPs was evaluated from the area of the hysteresis loop by hard type of cobalt MPs without bio-probes coating. Not only a calibration curve was founded for many hepatic cell carcinoma stages, but also microscale images verified the Ms increase due to magnetic protein clusters, etc. Hence, its wide populations in clinics could be expected.

## 1. Introduction

Nowadays, the occurrence of fatal diseases is increasingly occurring around the world such as cancer. Liver cancer ranked the third position of cancer death in worldwide according the WHO in which primarily are hepatocellular carcinoma (HCC) [1]. Early detection of disease is one of the most influence factors on the disease prognosis. The diagnosis stage of cancer mainly performs with radiological and blood serum examination. However, radiological diagnosis is manpower dependent, high cost and high exposure of radio- hazardous to both operator and patient than serum test in regular examination [2]. Disease diagnosis via the detection of biomarkers, such as so-called blood tests, was the most popular in clinics and bio-laboratories. Some biomarkers could be detectable directly and simply because their concentration was not too low, or their intrinsic characteristics were different from other materials in the biopsy mixture, such as the lipid in blood lipid [3] or urine protein [4]. Generally, the biomarkers were simple molecular without specific characteristics and in low concentration of ppm or ppb levels, and then artificial carriers with bio-probes mediated were used as the detect targets. 

Although the match of biomarkers and bio-probes, such as antibodies and antigens, were usually high specific and unique, the utilized artificial carriers were different electromagnetic-based materials. For example, radioimmunoassay utilized radiolabeled molecules in a stepwise formation of immune complexes, but some drawbacks were noted, such as the relatively short half-life of radiolabeled molecules and the possible safety hazards of radioisotope use [5]. Fluorescence immunoassay used the fluorescent label to attach to antibodies or antigens [6]. Its application was divided into liquid specimens, i.e., enzyme-linked immunosorbent assay [7], and solid tissue, i.e., immunohistochemistry [8]. Despite its greater biosafety and relatively longer lifetime than radioimmunoassay, it had some limitations, such as weak fluorescent intensity to detect in some solutions or thin tissue slices, time and manpower consumption, optical interference, and nonspecific bioconjugation.

**Figure 1:**
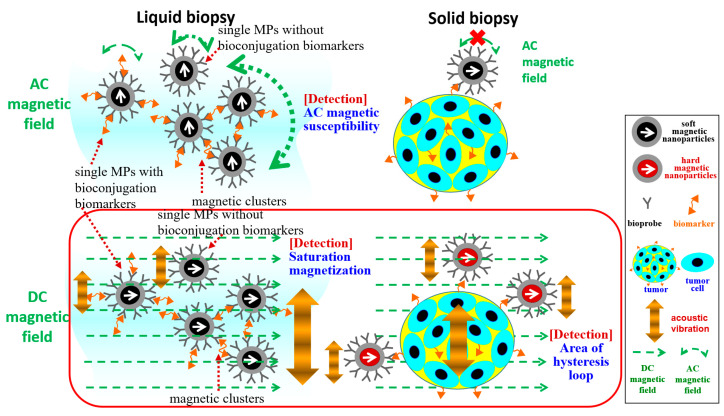
Mechanisms of different magnetic types of MPs as well as DC or AC Hs for molecular detections in liquid or solid biopsies. Here, the DC Hs accompany vibration excitation.

**Figure 2:**
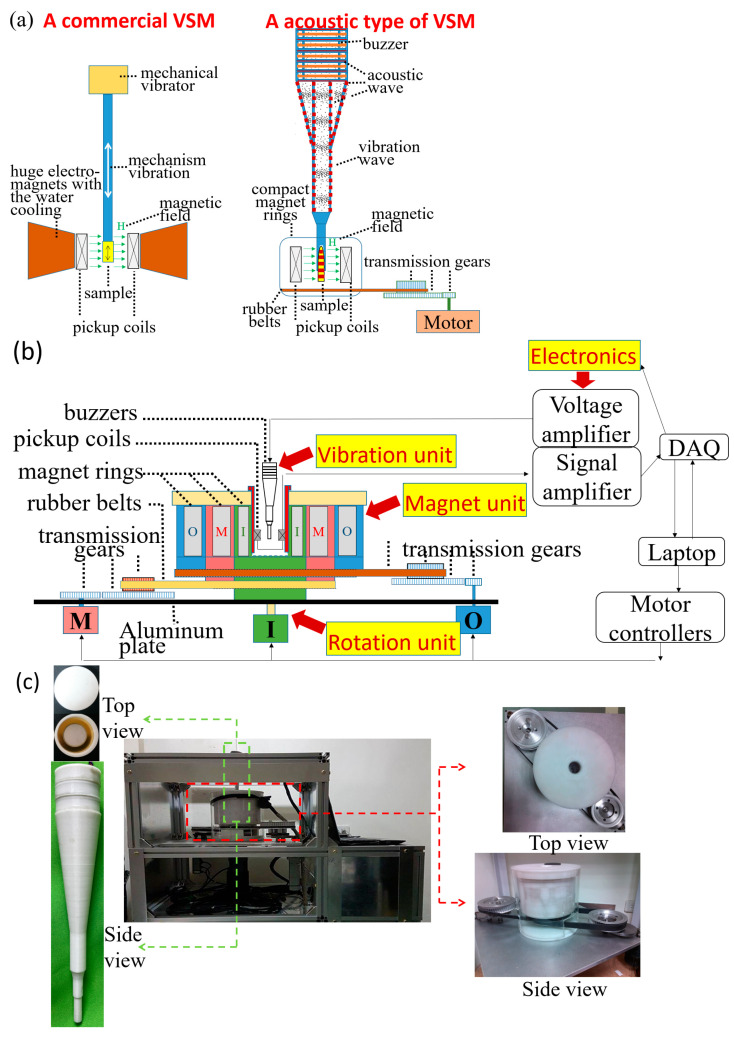
Acoustic type of VSM: (**a**) comparison of excitation and detection mechanisms with a general VSM, (**b**) complete scheme, and (**c**) photos.

Superior to mentioned electromagnetic-based materials, magnetic particles (MPs) have distinctive physical and chemical properties based on their structure, size, and shape. Moreover, MPs have extensive magnetic characteristics, such as the soft and hard magnetic types, i.e., smaller or larger magnetic excitation fields (Hs) for direct current (DC) saturation magnetization (Ms), decided by magnetic materials [9]. Indeed, the soft magnetic type of Fe_3_O_4_ MPs with the properties of low toxicity, biocompatibility, specific dispersion, and stability in solution became the most used in in vivo and in vitro diagnosis and treatment [10]. However, the hard magnetic types of Co MPs have more industry applications than biomedical ones, limited by the special green synthesis method [11]. For any type of MPs, the characterization of DC Ms was always limited by the well-known vibrating sample magnetometer (VSM) [12]. For example, the integration utility of Fe_3_O_4_ MPs and the VSM were verified for the diagnosis of common disease by screening the ppb levels in solutions of general disease biomarkers (the lower part of Figure 1) [13]. However, some intrinsic disadvantages of VSM mechanisms resulted in high financial cost, electrical power, and size, and, as such, few were produced (the left part of Figure 2a). First, the massive electromagnetic coil consumed a large amount of electric power and then required a water-cooling system. Second, the mechanical vibration mechanism required much power to generate the long, solid sample holder under low-frequency vibrations and required an additional alignment mechanism to limitedly keep sample positions against the environment’s influence. Here, vibration excitation existed in DC magnetization due to the Faraday induction theory.

Opposite to the DC magnetic excitation, alternating current (AC) magnetic excitation is another powerful method of MP-based diagnosis. Immunomagnetic reduction (IMR) became the representative magnetic immunoassay based on the AC magnetic susceptibility of Fe_3_O_4_ MPs [14,15], where the sensitivity of serum biomarkers was achieved in similar ppb levels of general disease by magnetic coils and common electronics with the VSM [14], and in the further ppt levels of Alzheimer’s diseases by the most sensitive magnetic sensor of the superconducting quantum interference device (SQUID) [15]. However, IMR was limited to the diagnosis of liquid solution samples and cannot be used for solid tissue samples, where the similar scanning SQUID biosusceptometry was made up by the expensive SQUID sensor. 

Given these limitations, diagnosis using current magnetic methodologies and instruments had neither high cost–performance value nor were they powerful for most samples of liquid and solid biopsies in clinics. Hence, this work proposed the versatile, accessible magnetic diagnostic platform based on the homemade acoustic type of VSM (Figure 2) and different magnetic types of MPs for suitable biopsies (Figure 1). These two parts were illustrated as follows. First, all the mechanisms illustrated the magnetic types of MPs and types of Hs depended on the biopsy types (Figure 1), although the molecular biomarkers of liquid or solid biopsies influenced these characterizations of labeled MPs.

(A) For liquid biopsies (the left part of Figure 1), the bioprobe-coated MPs were classified into three groups: single MPs without bioconjugation biomarkers, single MPs with bioconjugation biomarkers, and magnetic clusters. Fe_3_O_4_ MPs were suitable to align their magnetization direction along with the external Hs due to the superior AC magnetic susceptibility.
  ▪ Under AC Hs as the rapid rotation fields, single MPs without bioconjugation biomarkers kept the same AC magnetic susceptibility and then contributed no reduction in AC magnetic susceptibility. By contrast, single MPs or multiple MPs with bioconjugation happened to the reduction in AC magnetic susceptibility with the biomarker concentrations. Here, multiple MPs with bioconjugation formed magnetic protein clusters.
  ▪ Under DC Hs and vibration excitation, the larger MPs of the magnetic protein cluster contributed to the increased Ms.
    Between them, the detection based on the AC Hs was more sensitive to the liquid biopsy due to the minute difference of single MPs with bioconjugation biomarkers. In other DC Hs, the accompanying vibration mechanism could reduce the incubation time of bioconjugation in liquid biopsies due to the increased oscillation possibility compared with the vibration-free ones.
(B) For solid biopsies (right part of Figure 1), only the bounded MPs on solid tissue after washing unbounded MPs, i.e., the common step of any tissue stains, were remained. Without liquid floatation, MPs were difficult to “rotate” due to the random Brownian relaxation. Hence, instead of Fe_3_O_4_ MPs with high AC magnetic susceptibility, the hard magnetic type of MPs was proposed in this work.
  ▪ Under AC Hs, bounded MPs undoubtedly had very low AC magnetic susceptibility to detect the minute bioconjugation-induced variation [16].
  ▪ Under DC Hs, bounded MPs still aligned their magnetization along the direction of external DC Hs despite difficulties in rotation. Furthermore, the hard magnetic type of MPs contributed to the increase in the hysteresis area with the MP amount, i.e., the biomarker amount in tissue. Similarly, the relaxation methodology under the pulse sequence of Hs, as the short duration of DC magnetic fields, worked for free-bounded nanoparticles in some tissue by the measurement of relaxation time. Several limitations remained, such as the difficulty of measuring the long relaxation time when using high-sensitivity magnetic sensors, such as SQUID, against the noise influence [17].

Second, the versatile, accessible advantages of the homemade acoustic type of VSM (Figure 2b,c) resulted from the advances of two excitation devices, i.e., DC magnetic and vibration ones, compared with the general VSM (Figure 2a). The detection by the proposed methodologies for liquid and solid biopsies was verified in some tests. Furthermore, some molecular-scale images illustrated the bioconjugation hypothesis between molecular and bounded MPs. 

## 2. Results

### 2.1. Characterization of the Acoustic Type of VSM

The homemade acoustic VSM was characterized using two excitation devices, and then the detection sensitivity was determined. First, the distributions of DC Hs in the sample region around 2 × 2 cm were simulated. The H maximum and minimum values showed a quietly uniform distribution around 0.79 and 0 Tesla in (Figure S1a). However, the experimental results by the Gauss meter were around 0.6433 and 0.0001 Tesla. 

Second, the simulated acoustic pressure in the inner air space and tube walls of the acoustic vibrator varied with time (Figure S1b). At the start of no excitation, the whole pressure field was uniform and marked with the light green color of the zero level. In the following process of continuous sinusoid electrical signals to buzzers, the pressure distribution of one cycle varied with region and time, and was divided into three subphases, as follows:

▪ In Phase I from 0 to 148.2 μs, the major pressure increase happened in the upper air space of the central tube (the color variation from the light green to the yellow–green), and the minor one occurred in the lower air space of the central tube (from a dark blue color to a light blue one). By contrast, the pressure decreased in the upper neighbors to the central tube (the color variation from the green–blue color to the dark blue one) because their air was pushed into the central tube.
▪ In Phase II from 296.3 μs to 444.4 μs, the high-pressure air flowed majorly to the lower air space of the central tube (the color variation from the orange to the red) due to the nozzle effect and minorly to the upper neighbors of the central tube (from the light blue color to the light yellow one). By contrast, the pressure decreased in the upper air space of the central tube (the color variation from the light green color to the light blue one).
▪ In Phase III from 592.5 μs to 740.6 μs, the pressure developed inversely from Phase I to Phase II. For example, the pressure distribution at 444.4 μs, i.e., the end of Phase II, was viewed as the center of one cycle, and that at 592.5 μs, i.e., the start of Phase III, was symmetrically similar to 444.4 μs, i.e., the end of Phase II. Equally, that at 740.6 μs, i.e., the end of Phase III, was symmetrically similar with 0 μs, i.e., the start of Phase I.

Summarily, due to the continuous sinusoid excitation, no extra duration happened between the three phases of a vibration cycle. Phase I, II, and III could be simply and separately illustrated as the air import and the heating tube wall through the buzzle generation, the oscillation of a sample through pressured air and vibrated nozzle walls, and the air leakage and the vibration leaving the nozzle in addition to the repetition of the start step of Phase I.

### 2.2. Tests of AFP Standard Solutions

**Figure 3:**
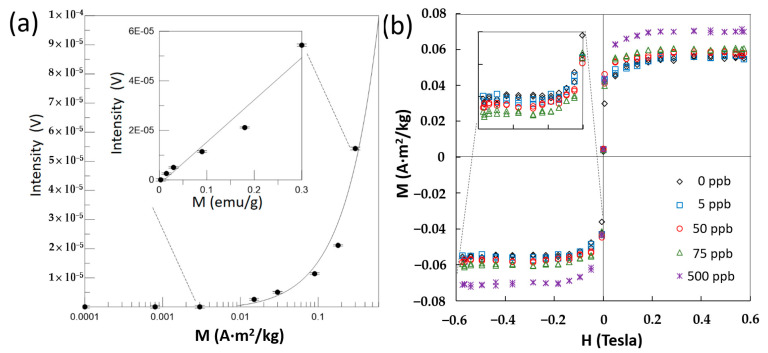
Study of magnetic sensitivity and tests of AFP standard solutions: (**a**) Different concentrations (Ms = 0.3, 0.18, 0.09, 0.03, 0.02, 0.003, 0.0008, 0 A·m^2^/kg) of AFP reagent in 100 µL for sensitivity tests; (**b**) M–H curves of the mixtures, composed of different AFP concentrations (0, 5, 50, 75, 500 ppb) of AFP standard solutions in 60 µL and the AFP reagent (0.3 A·m^2^/kg) in 40 µL.

The first test was to study of the magnetic sensitivity of this acoustic type of VSM by the AFP reagents in different concentrations (0.3, 0.18, 0.09, 0.03, 0.02, and 0.003 A·m^2^/kg) and 100 µL. The Ms of M–H curves was linear between the detected voltage and the M as the inset of Figure 3a. However, this linear fit changed its shape due to the variation of M axial from the log-scale to linear scale while the detected intensities of two ultra-small M (0.0008, 0 A·m^2^/kg) were added to verify that the lowest detectable Ms was 0.003 A·m^2^/kg. The zero concentration was plotted as 0.0001 A·m^2^/kg because no zero value was observed on the log-scale axes. 

After mixing AFP standard solutions of different concentrations (0, 5, 50, 75, and 500 ppb) and 60 μL with the same AFP reagent of 0.3 A·m^2^/kg and 40 μL, the post bioconjugation M–H curves are shown in Figure 3b. Here, the AFP reagent was based on the soft magnetic type of Fe_3_O_4_ MPs for liquid biopsies. For the highest AFP concentration of 500 ppb, the absolute Ms value of the M–H curve was the largest. For other AFP concentrations, their differences of M–H curves were compared in the inset. The absolute M increased with the AFP concentrations, except for the seldom distinguished concentrations of 0 and 5 ppb. Moreover, the Ms of all concentration samples at zero H were commonly around 0.003 A·m^2^/kg due to the nonperfect unbalance between the two sub-sensing coils of the pair of sensing coils. 

### 2.3. Imaging the Bioconjugation Mechanism

**Figure 4:**
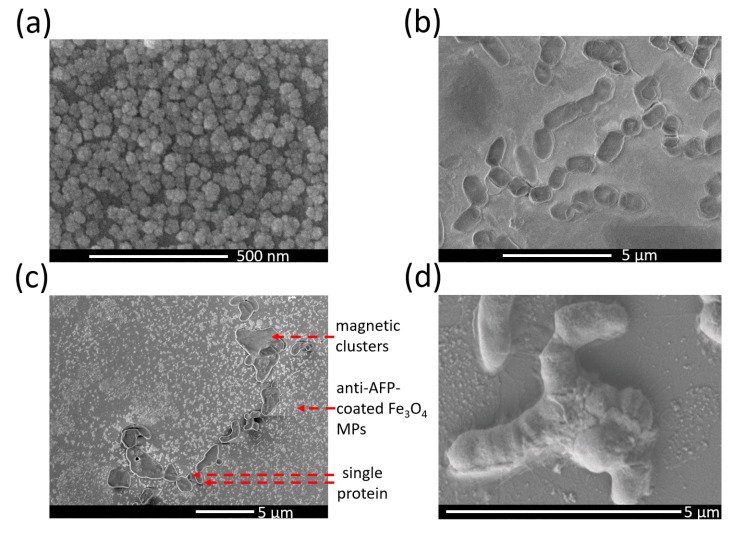
SEM images of MPs and molecules: (**a**) pure bioprobe-coated MPs of the utilized AFP reagent; (**b**) pure AFP proteins; (**c**) post-bioconjugation materials including unbounded and bounded MPs, single proteins, and protein clusters; (**d**) focus view of the post-bioconjugation protein clusters.

The scanning electron microscope (SEM) images showed the material appearance of pre- and post-bioconjugation in the tests of 50 ppb standard solution (Figure 3). For example, anti-AFP-coated Fe_3_O_4_ MPs (Figure 4a) and AFP proteins (Figure 4b) were shown at around 50 nm and 1 μm, respectively. Figure 4c shows all the materials of post-bioconjugation, i.e., anti-AFP-coated Fe_3_O_4_ MPs, single AFP proteins, and magnetic protein clusters in different sizes. Here, single AFP proteins may have zero or several targeted anti-AFP-coated Fe_3_O_4_ MPs. However, due to the bioconjugation of AFP antibodies on single MPs to different AFP antigen proteins (Figure 4c,d), magnetic clusters were composed of few AFP proteins and several targeted anti-AFP-coated MPs, as depicted in the focus view of one magnetic protein cluster around 2 μm (Figure 4d). 

### 2.4. Blood Tests 

The AFP concentrations of the subject serum were classified into three types by radioimmunoassay (RIA) methods: three samples of normal subjects (2.75, 3.78, and 15.00 ppb, named as C-1, C-2, and C-3, respectively), two samples of low risks (83.0 and 151.2 ppb, named as L-1 and L-2, respectively), and two samples of high risks (2356.33 and 26678.2 ppb, named as H-1 and H-2, respectively). Here, the AFP level of the normal subject group was indeed lower than the screen criterion of 20 ppb, and those of the LR and HR series were definitely discriminated by the strong suggestion level of 500 ppb for HCC [1,2]. Based on the IRB-approved blood test (IRB Number 201105006RC, National Taiwan University Hospital, Taipei, Taiwan), these AFP concentrations were diagnosed using radioimmunoassay at the Department of Laboratory Medicine, National Taiwan Hospital. Furthermore, the utilized AFP reagents (MF-DEX-0060, MagQu, New Taipei, Taiwan, Republic of China) composed of dextran-shelled Fe_3_O_4_ MPs belonged to the soft magnetic type of MPs, had AFP antibody coating, and were dispersed in phosphoryl buffer solution (PBS). 

**Figure 5:**
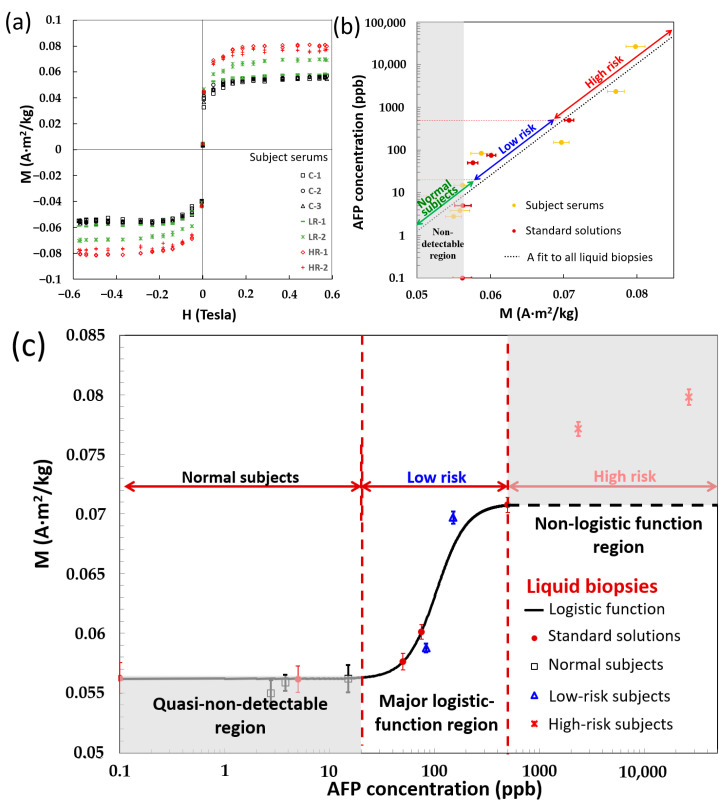
Different tests of liquid biopsies: (**a**) blood tests by only the acoustic type of VSM. M–H curves of subject serum. C, LR, and HR indicate normal, low risk, and high risk, respectively. (**b**) Fit line between AFP concentrations and Ms for all liquid biopsies (AFP standard solutions and subject serum). (**c**) Calibration line between AFP concentrations and Ms for only AFP standard solutions. Here, AFP concentrations of subject serums were obtained by RIA.

For the mixture of AFP reagent of 0.3 A·m^2^/kg and 40 μL and serum samples of 60 μL, seven M–H curves of subject serums showed superparamagnetism without hysteresis loops (Figure 5a) similar to AFP standard solutions (Figure 3b). Moreover, three M–H curves (C-1, C-2, and C-3) in black colors were too close to distinguish because their AFP concentrations were lower than the screen criterion of 20 ppb. Consequently, the Ms of four M–H curves of LR and HR series in green and red colors were significantly apart from the black colors, and their difference levels increased with AFP concentrations, i.e., the HCC risk stage. 

From all liquid biopsies (AFP standard solutions and subject serums), the relationship between the log-scale AFP concentration and the Ms showed a good fit with a high coefficient of determination (R^2^) of around 88.3% (Figure 5b). This result indicated the good agreement of AFP concentrations between subject serums and AFP standard solutions. Moreover, the Ms of three normal subject serums (2.75, 3.78, and 15.0 ppb in yellow colors) and two AFP standard solutions (0 and 5 ppb in brown dots) were almost around 0.058 A·m^2^/kg, defined as the boundary of the gray undetectable region. Hence, by the intersection point of this fit line and a vertical line through the 0.058 A·m^2^/kg, the detectable minimum of an acoustic type of VSM could be determined at 8 ppb. Here, AFP standard solutions of 0 ppb were plotted at 0.1 ppb because no zero value was observed in the log-scale. In addition, the fit was divided into three levels of normal, low risks, and high risks according to the screening criterion of 20 ppb and the moderate HCC stage of 500 ppb. 

Although the fit line (Figure 5b) statistically illustrated that the AFP concentrations of subject serums were close to the AFP standard solutions, the satisfactory performances demonstrated the accuracy of the used RIA technology. To find the calibration line of the acoustic type of VSM, only AFP standard solutions were used based on the logistic function of the MP-related immunoassay model (Figure 5c) because the lowest detection and highest saturation levels happened based on the conjugation between biomarkers and bioprobe-coated MPs [9,10]. Differently from the fit in Figure 5b, this calibration line was classified into two parts. The first was the horizontal lowest Ms level at the zero AFP concentration until the first inflection point of 20 ppb, and the second was a major part of logistic function for the low-risk level until the second inflection point of 500 ppb. Consequently, a horizontal dot line was prolonged from this top saturation Ms. 

Consequently, the data points of all liquid biopsies were plotted by their Ms in Figure 5c. Two AFP standard solutions (0 and 5 ppb in red dots) and three normal subject serums (2.75, 3.78, and 15.0 ppb in black colors) were close to the first part of the calibration line, similar to Figure 5b. This region was named as the stage of normal subjects or the quasi-nondetectable region; three AFP standard solutions (50, 75, and 500 ppb in red dots) and two low-risk subject serums (83.0 and 151.2 ppb in blue colors) were also near the second part of the calibration line. This region was named as the low-risk stage of HCC. These mentioned data and the entire logistic function covering these two HCC stages had a better R^2^ of 96.3% than the fit of 88.3% in Figure 5b. However, two high-risk subject serums (2356.33 and 26678.2 ppb) were reasonably far from the extension dot line and were not constructed from any data point of AFP standard solutions. This region over the horizontal dot line was named as the high-risk stage of HCC or the non-logistic function region. Hence, this chart (Figure 5c), composed of this logistic function and the horizontal dotted line, was useful for the screening of regular healthy individuals and the confirmation of some key HCC stages using this acoustic type of VSM. The limited utility of the first two parts of the logistic function was the same as in other MP-based immunoassays [9,10].

### 2.5. Tests of Solid Biopsy

**Figure 6:**
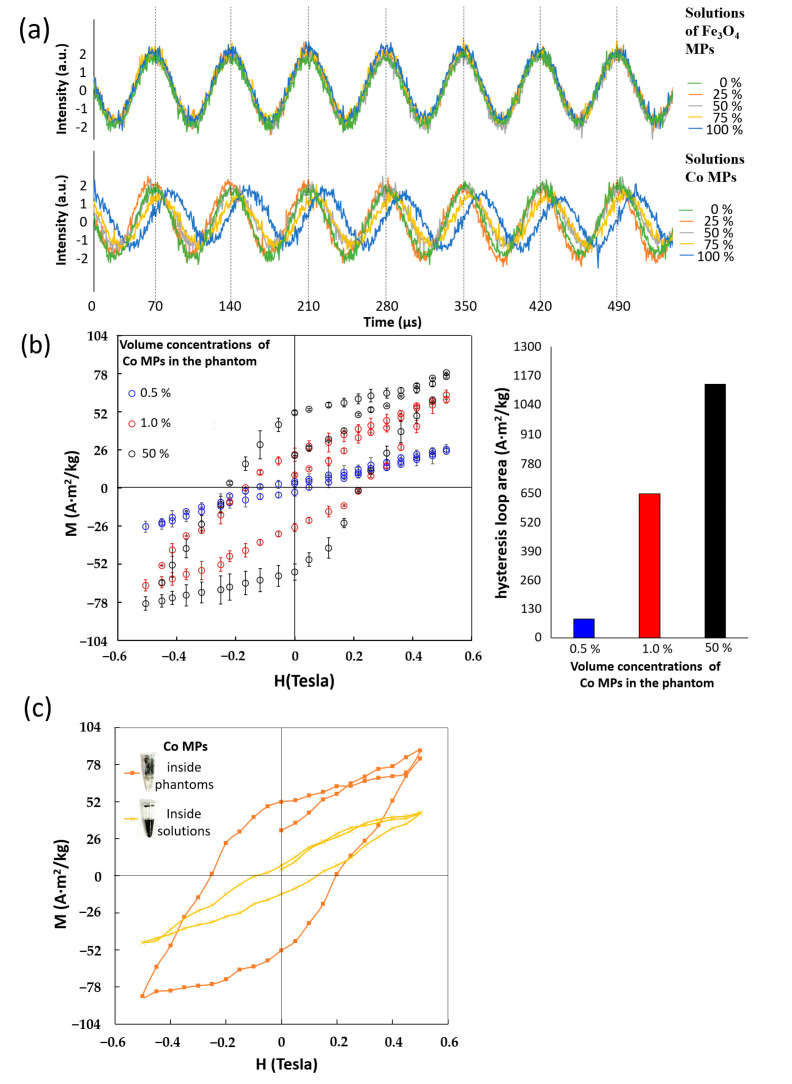
Solid biopsy tests of reference and experiment groups: (**a**) The reference group was the mixture of water as the vibration propagation material, Fe_3_O_4_, and Co MPs under the AC H of 0.065 Tesla at 1.4 kHz. The waveform acquisition of MPs in volume concentrations (0%, 25%, 50%, 75%, and 100%). (**b**) The experimental group was the mixture of the phantom as the vibration propagation materials and only Co MPs under the DC H by the acoustic type of VSM. The hysteresis loop acquisition of MPs in volume concentrations (0.5%, 1%, and 50%). (**c**) Comparison of hysteresis loops between one sample of Co MPs at the same volume concentration of 50% from these two groups.

MPs without bioprobe coating and surfactants were mixed with water and the phantom of different vibration propagation materials for the reference and experiment groups. In the reference group, the two mixtures were water and soft magnetic types of Fe_3_O_4_ MPs or hard magnetic types of Co MPs in volume concentrations of 0%, 25%, 50%, 75%, and 100% in microtubes under the AC H. For the sinusoidal waveform of detected voltages with time (Figure 6a), Fe_3_O_4_ MPs had no dissimilarities between different MP volume concentrations; Co MPs showed a similar phenomenon in lower volume concentrations of 0%, 25%, and 50% (green, orange, and gray labels, respectively), but the separation of sinusoid profiles in higher volume concentrations of 75% and 100% (the yellow and blue labels) was apparent compared with those in the lower ones. 

In the experimental group, the phantom was simulated as the real tissue with biotargets and the vibration propagation material and was mixed with Co MPs belonging to hard magnetic type of MPs for a reliable, uniform MP distribution. Consequently, the hysteresis loops of Co MPs in volume concentrations of 50%, 1.0%, and 0.5%, labeled with black, red, and blue colors, respectively, by the acoustic type of VSM, showed the largest, middle, and seldom open holes, respectively (left of Figure 6b). Here, the hysteresis loop was defined as a closed loop between two symmetric points in the positive and negative Hs of an M–H curve under the DC H. The area of the hysteresis loop increased with volume concentrations. Finally, between the same volume concentration of 50% Co MPs in the phantom and water, the loop area and loop close point intensities of hysteresis loops were apparently large and small (Figure 6c), respectively. Here, the loop close point intensity was defined as the measured intensity around the DC Hs of ±0.5 Tesla.

## 3. Discussion

For the proposed acoustic type of MPs, two simulation excitation results of H distributions in a sample region and the pressure inside the acoustic vibrator showed reasonable concepts. For example, the simulated H distribution (Figure S1a) had a small difference from the experimental results only in the maximum H value. The real H distribution of real long-bar magnets in the magnet rings was not uniform as there was only one ideal value of the simulation hypothesis. Moreover, the real H variation between +0.6 T and −0.6 T was enough to achieve the Ms of soft magnetic types of MPs for liquid biopsies of magnetic-based immunoassay and to close the hysteresis loop of hard magnetic MPs for solid biopsies. In addition, the simulated pressure distribution of one cycle (Figure S1b) illustrated how the acoustic wave of air space and the vibration wave of acoustic vibrator walls separately propagated to oscillate the whole microtube container, and then penetrate inside liquid or biopsy samples. Moreover, as the sinusoidal waveform of the input electrical signal, the pressure in the lower air space of the acoustic vibrator achieved the maximum value needed to vibrate a sample tube at the end of Phase II and became the minimum at the end of Phase III and the start of Phase I. The total time of one simulation cycle was 740.6 μs, equal to 1.35 kHz, which was in good agreement with the electrical signal at 1.4 kHz.

In the experimental sensitivity of the soft magnetic type of MPs, the detectable range was 3 × 10^−4^ to 3 × 10^−2^ emu, obtained from the product of the detectable Ms of 0.003 A·m^2^/kg (Figure 3a) and the volume of 100 µL, similar to 1 × 10^−4^ to 1 × 10^−2^ emu in other works [18]. A similar sensitivity could be achieved by this proposed versatile, accessible magnetic platform. The improvements in volume, weight, and power consumption were indeed due to the upgrade of these two excitation mechanisms of magnetic and vibrational aspects. Moreover, the magnetic immunoassay for liquid biopsies was undoubtedly workable because the same utility of soft magnetic type of MPs made the initial DC Hs at the low Ms. 

Despite different sizes, these MPs, magnetic protein clusters, and proteins (Figure 4c) were much smaller than the excitation acoustic wavelengths in order to absorb the most vibration energy. Hence, the excitation vibration had no damaging risks for any material but propagated through the solvents to accelerate the oscillation of any materials. It helpfully shortened the incubation time for the magnetic protein clusters, i.e., the bioconjugation between MPs and proteins. The secondary acceleration mechanisms were the effects of magnetic force on MPs for growing magnetic protein clusters, vertical to the vibration force. Under these vertically external forces of acoustic vibrating force and magnetic attractions, the inspections and incubation mechanisms for the acoustic type of VSM and magnetic protein clusters could be achieved.

The single magnetic protein cluster with more AFP-coated Fe_3_O_4_ MPs possessed a larger M than one protein with AFP-coated Fe_3_O_4_ MPs, i.e., few proteins in AFP standard solutions of 5 ppb or serums of normal subjects, and then enlarged the M of the mixture. Owing to the lack of residue magnetism difference and the lack of hysteresis introduction, magnetic protein clusters were the same when superparamagnetic and M-enhanced with their components of anti-AFP-coated Fe_3_O_4_ MPs. The measured magnetic signal by this acoustic type of VSM type not only simply increased with larger targeted proteins than MPs, but also was independent of MP sizes. It was superior to other magnetic immunoassays, for example, the IMR technology, with only the measurement mechanism without the incubation mechanism and the MP-size-dependent magnetic relaxation technology. However, the single MP with bioconjugated biomarkers did not contribute to the M increase by the acoustic type of VSM but significantly resulted in a reduction in AC susceptibility in IMR. Hence, the IMR technology was more sensitive in the low detection limitation of 3 ppb than 8 ppb in this work by the same AFP reagents and standard solvents [19,20] despite dissimilar operating conditions with different inspection instruments.

Here, the M of the magnetic protein cluster was larger than that of the single protein, and this finding agreed with other works that the excitation fields are influenced by the size of the target molecule [21]. Hence, the tests of standard solutions and subject serums were focused on only AFP proteins. For example, the Ms values of AFP standard solutions in low ppb levels were undistinguishable (Figure 3b), but the Ms values of normal subjects were still lower than those of the low-risk and high-risk groups (Figure 5). Based on the logistic function in this paper, the calibration curve of AFP concentration was between 8 and 500 ppb as the lowest and highest cut off points, respectively (Figure 5c). Consequently, it was workable for the early diagnosis of HCC-related diseases through serum samples. 

Generally, the well-known unit of ppb of molecular concentration is used for the biomarker concentration in the AFP standard solution. Conversely, the AFP antibody coating in AFP reagents are not necessarily known. Hence, the concentration of AFP reagent is focused on the concentration of MPs with AFP antibody coating. The general concentration unit of ppb is suitable for only the MP concentrations, but not for MP types, especially the two utilities of soft and hard magnetic types of MPs in this work. Conversely, the unit of magnetization (A·m^2^/kg) is able to describe the total level of the magnetic type and amounts of MPs with an AFP antibody coating. For example, the concentration of anti-AFP-coated Fe_3_O_4_ MPs influenced the saturation magnetization Ms of the entire AFP reagents (Figure 3a), and the Ms increased after the bioconjugation with biomarkers (Figure 3b and Figure 4a). Hence, the unit of magnetization is suitable for the AFP reagents in this work and other similar MP works [13,19,20].

This proposed acoustic type of VSM and the IMR analyzer based on the DC Ms and AC susceptibility showed an ultra-low concentration of AFP molecular similar to that of the conventional ELISA kit due to the superior anti-interference property of unique DC and AC magnetization variation of the soft magnetism of bioconjugation MPs [13,19]. Here, the AC susceptibility technology utilized the mixed frequency of two AC Hs rather than the single frequency of one AC H [22]. The reason was that the excitation signal was always a major part of the detected signal at the excitation frequency. The detected signal based on AC magnetization occupied a few parts. Hence, the same detected intensity of all MP concentrations was zero or one regardless of soft or hard magnetic types of MPs (Figure 6a). 

All MP concentrations, except zero, were too high to dissolve in water solutions because the saturation concentration was always small for whatever magnetic type of MPs and the surfactant coating that existed on MPs or not. In addition, the precipitated MPs under the water solution, for the example of 50% Co MPs (the lower one in the inset of Figure 6c), rather than resulting in suspended MPs, expressed the inconsistent waveforms at ultra-high concentrations of 75% and 100% for only Co MPs (Figure 6a). The inconsistent waveforms under the AC H indicated that the magnetization signal did not follow the excitation signal, resulting in the same physical meaning with the area of a hysteresis loop under the DC H [23]. The AC magnetic susceptibility technology was sensitive to only the AC magnetic susceptibility of free rotation MPs based on free rotation mechanisms [21] and not to inconsistent waveforms between the excitation and detection signals of precipitated MPs. 

Similarly, for the hard magnetic types of MPs, the hysteresis loop under DC H had a better resolution in MP concentration (at least from 0.5% to 50%) than the waveform consistence under the AC H (necessarily larger than 50%), as shown in Figure 6a,b. Here, the phantom mixed with non-bioprobe MPs was used to simulate the bounded MPs in tissue for the immunohistochemistry. 

The elastic phantom was tissue promoted to vibrate MPs under another acoustic vibration, different from the DC H. Hence, for Co MPs in the same concentration of 50%, the area of a hysteresis loop of the uniform distribution (the upper one in the inset of Figure 6c) was much larger than the precipitation under a thick water layer. Moreover, the Ms of soft magnetic type of MPs, such as Fe_3_O_4_ MPs, was generally much lower than that of the hard ones [24], and the M detection was always too weak to rely on the aforementioned high-cost, complicated SQUID-based technology [15,20]. For the presented high MP concentrations, it could be explained in two aspects of the wide-enough inspection ranges and the experience of the ex vivo general stain, rather than the target after the injection. First, the inspection range should cover more parts of all disease phases in order to achieve the best levels of larger than the whole diagnosis demand. Second, the tumors of solid biopsies are formed by lumps of tumor tissue, i.e., full of tumor cells. Consequently, the ex vivo general stain, such as the H&E stain or Prussian stain, always showed lots of dye remaining on all the entire slices, as opposed to only some dye or molecules “targeted” on the same lesion tissue after the dilution through the injection into the blood circulation of living animals or subjects. Hence, the proposed magnetic stain based on Co MPs instead of these dyes of general stains expectedly expressed more stained cells in tissue in a shorter time due to the enhanced penetration ability and greater oscillation probabilities under the acceleration of magnetic force [25]. Besides, the large uncertainties for 50% “volume concentration” of Co MPs in the phantom resulted from the phenomenon that the rotation of strong magnetic force from different magnetic rings made high-concentration and non-bioconjugation Co MPs move insides the phantom. Unlike the general materials with non-movable magnetic compositions, the higher-concentration magnetic compositions in the phantom easily expressed the higher movable possibilities insides the phantom. 

## 4. Materials and Methods

### 4.1. Homemade Acoustic Type of Vibration Sample Magnetometer 

**Figure 7:**
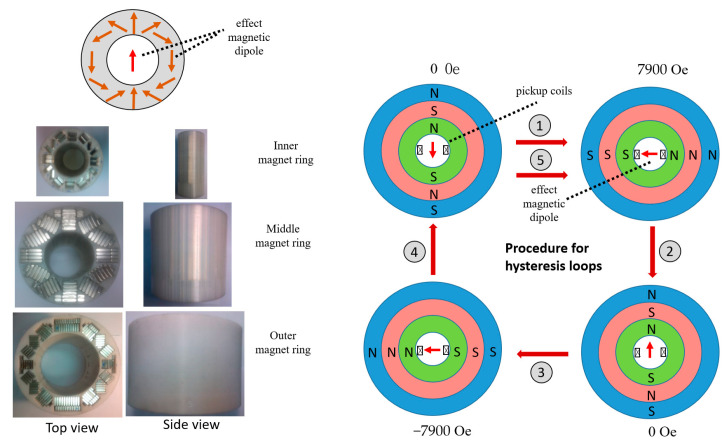
Photos of magnet rings for the construction scheme of one net H (**left**) and the modulation of H strengths for an M–H curve (**right**).

The proposed acoustic type of VSM in this work was studied as follows (the right part of Figure 2a,c). First, in the detection aspects, the utility of first-order pickup coils as the magnetic sensors was the same as the general VSM (left part of Figure 2a). Second, in the excitation aspects, the rotational and compact magnet rings and simple acoustic vibrator (Figure 2b) as magnetic and vibration excitation devices separately the huge electromagnet and the mechanical vibration motor, respectively, solving the two disadvantages. In particular, the magnet ring, i.e., one of the Halbach arrays [26], was composed of several long-bar and strong-strength magnets in designed positions that achieved the effective strength variation of the DC magnetic fields in the detection direction, i.e., the direction of the cross line between the pickup pair (Figure 7). Consequently, each magnetic ring was marked with dipoles S or N due to the single effective magnetic field, and three magnet rings with different effective single magnetic directions resulted in the net magnetic excitation field in the detection direction. Thus, the huge electromagnet with ultra-large power consumption could be economically replaced by the group of magnetic rings and the rotation mechanism, including rubber belts, transmission gears, and motors without large power consumption (Figure 2b). 

For a typical hysteresis loop, the magnetic excitation fields in the detection direction increased from 0 Tesla to the positive maximum value of 0.79 Tesla (step 1 in Figure 7), then reversely (step 2 in Figure 7) from 0 Tesla to the negative maximum value of −0.79 Tesla (step 3 in Figure 7), and then reversely (the step 4 in Figure 7) again from 0 Tesla to the positive maximum value of 0.79 Tesla (step 5 in Figure 7). Here, the net magnetization in the detection direction, such as the maximum and minimum values of 0.79 and 0 Tesla, respectively (Figure S1a), and the acoustic wave propagation inside the acoustic vibrator could be simulated by using COMSOL Multiphysics software (version 5.6, COMSOL, Inc., Burlington, MA, USA), as shown in Figure S1b. The net magnetic fields of the simulated positive maximum and zero value were calibrated by the Gauss meter (5180, F. W. Bell Inc., Milwaukie, OR, USA). However, the acoustic pressure of the inner air space and tube wall were just simulated and not measured experimentally.

Moreover, in the acoustic type of vibration excitation, the utilized acoustic vibrator with ultra-low power consumption was consulted in a simple structure of the top four buzzers in series, a sonic tube in a nozzle, and a sample region in the nozzle end (Figure 2). The continuous sinusoid wave with 1.35 kHz drove these buzzers. Hence, the acoustic type of VSM was simply constructed with electronics and these mentioned units (Figure 2). The entire system and the connected computer were as compact as the tabletop (Figure 2c).

### 4.2. Soft Magnetic Types of Fe_3_O_4_ MPs for the Diagnosis in Two Liquid Biopsies

The utilized AFP reagents (MF-DEX-0060, MagQu, New Taipei, Taiwan, Republic of China) were composed of dextran-shelled Fe_3_O_4_ MPs and belonged to the soft magnetic type of MPs, had AFP antibody coating, and were dispersed in phosphoryl buffer solution (PBS). In the sensitivity test, the AFP reagents in a microtube (Microtube, Deltalab, Barcelona, Catalonia, Spain) had different concentrations (0.3, 0.18, 0.09, 0.03, 0.02, and 0.003 A·m^2^/kg) and measured 100 µL. In the tests of liquid biopsies, the mixture ratio of the same total 100 μL was 40 μL of the AFP reagent at 0.3 A·m^2^/kg and 60 μL of sample solutions.

One type of liquid biopsies was the AFP standard solutions of AFP antigens (EA502-Q1053; EastCoast Bio, North Berwick, ME) in the PBS. In addition to the PBS as the AFP solution of 0 ppb in concentration, the four other test solutions of 500, 75, 50, and 5 ppb in concentrations were obtained by diluting the obtained standard solution.

The other type was seven patient serums based on the IRB-approved blood test (IRB Number 201105006RC, National Taiwan University Hospital, Taipei, Taiwan). These seven serums were also diagnosed as 15.00, 3.78, 2.75, 83, 151.2, 2356.33, and 26678.2 ppb, respectively, with the radioimmunoassay in the Department of Laboratory Medicine, National Taiwan Hospital.

### 4.3. Imaging the Bioconjugation between Molecular and Bioprobe-Coated MPs

The morphologies of Fe_3_O_4_ MPs with anti-AFP antibody coating, and the bioconjugation cluster of AFP antigens and AFP-antibody-coated MPs were inspected with field-emission SEM (S-4700, Hitachi, Tokyo, Japan). For the sample preparation, the AFP antibody-coated MPs were dispersed in ultra-diluted solvent in the ratio of 1:50 at first and dried on a carbon tap (Nisshin-731, Agar Scientific, Stansted, UK) under vacuum at room temperature for 48 h. 

### 4.4. Hard Magnetic Types of Co MPs for the Test of Solid Biopsies

In the tests of the solid biopsy, the bioprobe conjugation exhibited more non-repeatability than the liquid biopsy due to there being no solution convention mechanism for the collision between biotargets and bioprobes. Hence, MPs without bioprobe coating and surfactants were utilized. Moreover, two groups of water and phantom as the vibration propagation materials were tested under the AC H and the DC H as well as the acoustic vibration, respectively. The mixtures of the reference group were the water as the vibration propagation material and Fe_3_O_4_ MPs (637106, Sigma-Aldrich Corp., St. Louis, MO, USA) or Co MPs (HK8555, Gredmann Taiwan Ltd., New Taipei, Taiwan) in MP volume concentrations of 0%, 25%, 50%, 75%, and 100% in microtubes. Here, the filled volume of MPs was obtained by the sequence process of filling the same volume of water, drawing the marker line, and drying out in advance. The MP volume concentration was defined as the volume ratio of MPs over the mixture of MPs and water, and we tried to illustrate different magnetic responses due to different magnetic types of MPs (Figure 6a). The particle sizes of the obtained Fe_3_O_4_ MPs and filtered Co MPs by a 10,000-mesh net were 50–100 nm and around 853 nm, respectively. The applied AC H was 0.065 Tesla at 1.4 kHz by a homemade coil, and the waveform detection was carried out using the oscilloscope (TBS1000C, Tektronix, Beaverton, OR, USA). This reference group was used to verify the hysteresis loop under the DC H by another view of the inconsistent waveforms under the AC H. 

In the experimental group, the mixtures were made of the liquid phantom and Co MPs belonging to hard magnetic type of MPs in fine volume concentrations of 0.5%, 1%, and 50%, and cooled to form the solid one in a microtube. Here, the phantom simulated the real tissue with biotargets the reliable and uniform MP distribution inside the vibration propagation material. The phantom was synthesized as follows. The stir bar at 300–350 rpm was used to stir the deoxygenated water of 12 mL and the filtered protein of 50 mL from the egg white in the beaker. The mixture turned white initially but became transparent after the addition of 19:1 acrylamide/Bis solution (a3449, Sigma-Aldrich Co., Ltd., St. Louis, MO, USA) of 30% in solution. Under the stirring condition, the order of three added chemicals was glycerol anhydrous (56-81-5, Sigma-Aldrich Co., Ltd., St. Louis, MO, USA) of 4.5 mL, ammonium persulfate solution (UN1444, Mallinckrodt Baker Inc., Phillipsburg, NJ, USA) of 10% in volume concentration and 0.5 mL, and TEMED (N, N, N′, N′-Tetramethylethylenediamine) (A12536, Alfa Aesar Co., Ltd., Heysham, UK) of 0.05 mL. After the achievement of a uniform state, the stirring was turned off, and the synthesized solutions were poured into containers and solidified, accompanied with the manual operation of gas extraction.

## 5. Conclusions

The versatile and accessible magnetic diagnosis platform was proposed for the clinical examination of liquid and solid biopsies by the soft type of Fe_3_O_4_ MPs and the hard type of Co MPs through the saturation magnetization Ms and the area of a hysteresis loop in an M–H curve, respectively. The different magnetic mechanisms were explained in schemes. In particular, the Ms increase with the molecular concentrations was verified by the bioconjugation for magnetic clusters in SEM images. Furthermore, a developed calibration curve for some key hepatic cell carcinoma stages theoretically agreed with the well-known logistic function, the same as in another MP-based immunoassay. Similarly, the wide detectable range of MP in the phantom mixture simulated as bounded MPs in tissue was also experimentally verified. The most important finding was the innovative acoustic type of VSM based on rotational and compact magnet rings and a simple acoustic vibrator to avoid the use of a huge electromagnet with ultra-large power consumption. These findings are expected to have applications in clinics.

## Data Availability

The data and materials that were analyzed in the current study are available from the corresponding author upon reasonable request.

## Supplementary Materials

The following supporting information can be downloaded at https://www.mdpi.com/article/10.3390/ijms241210363/s1.
