# Supplementary material for: Versatile and Accessible Magnetic Diagnosis Platform with Different Types of Magnetic Particles for Liquid and Solid Biopsies
Source: Int J Mol Sci. 2023 Jun 20;24(12):10363. doi: 10.3390/ijms241210363 (PMC10299023; doi:10.3390/ijms241210363)
Supplement: Supplementary file 1 [file ijms-24-10363-s001.zip › ijms-2394689-supplementary.pdf]

International Journal of
Molecular Sciences

MDPI

Supplemental Materials

The software COMSOL for Multiphysics Simulation was used to replicate the H variation with the rotations of three magnetic rings (Figure S1a) and the pressure variation with acoustic propagation after its generation from the top planar (Figure S1b).

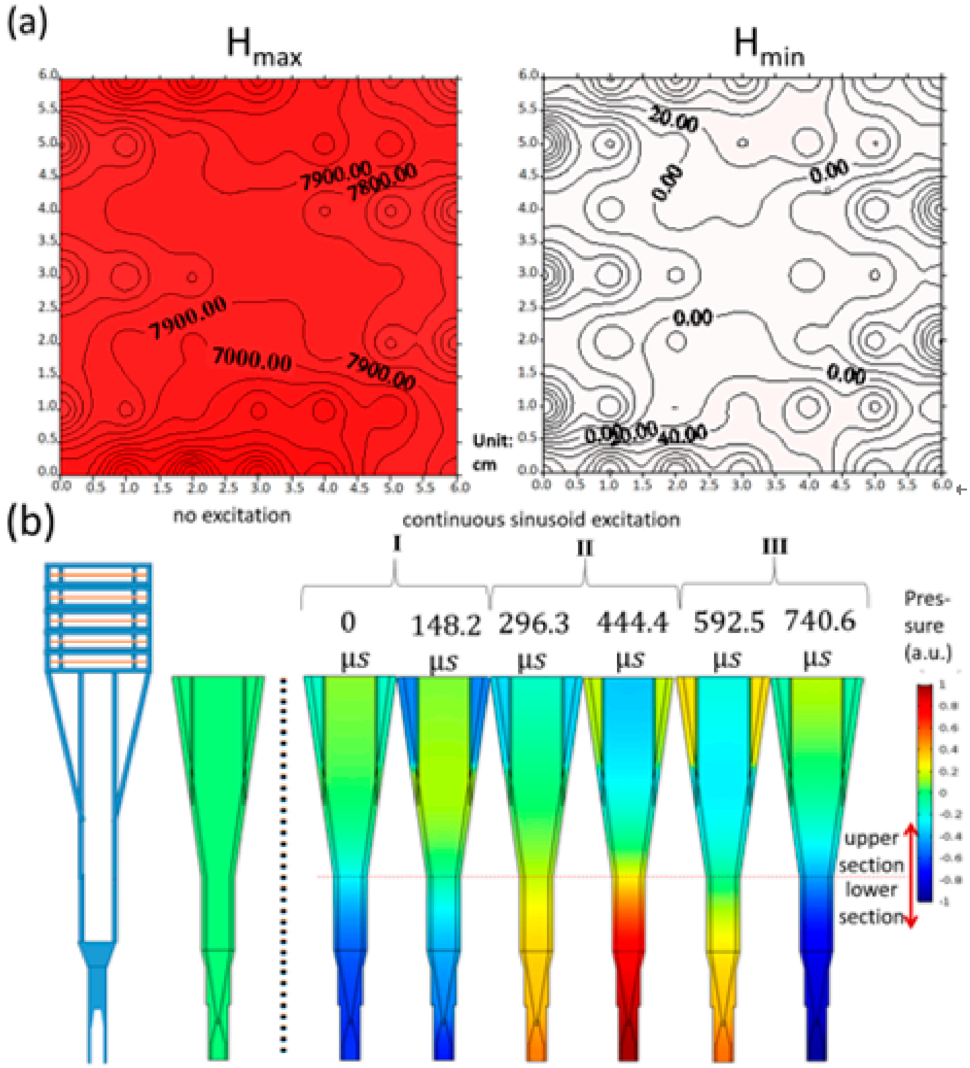

Figure S1. Simulated data of magnetic and acoustic excitation devices: (a) uniform magnetic field of H_{min} and H_{max} at the sample region between the pair of pickup coils;(b) acoustic vibrator and simulation of acoustic wave propagation
